# Pathological Mechanism of a Constitutively Active Form of Stromal Interaction Molecule 1 in Skeletal Muscle

**DOI:** 10.3390/biom11081064

**Published:** 2021-07-21

**Authors:** Ji Hee Park, Seung Yeon Jeong, Jun Hee Choi, Eun Hui Lee

**Affiliations:** 1 Department of Physiology, College of Medicine, The Catholic University of Korea, Seoul 06591, Korea; package0329@catholic.ac.kr (J.H.P.); tjdus0560@catholic.ac.kr (S.Y.J.); junhee@catholic.ac.kr (J.H.C.); 2 Department of Biomedicine & Health Sciences, Graduate School, The Catholic University of Korea, Seoul 06591, Korea

**Keywords:** skeletal muscle, STIM1, hyper-SOCE, cytosolic Ca^2+^

## Abstract

Stromal interaction molecule 1 (STIM1) is the main protein that, along with Orai1, mediates store-operated Ca^2+^ entry (SOCE) in skeletal muscle. Abnormal SOCE due to mutations in STIM1 is one of the causes of human skeletal muscle diseases. STIM1-R304Q (a constitutively active form of STIM1) has been found in human patients with skeletal muscle phenotypes such as muscle weakness, myalgia, muscle stiffness, and contracture. However, the pathological mechanism(s) of STIM1-R304Q in skeletal muscle have not been well studied. To examine the pathological mechanism(s) of STIM1-R304Q in skeletal muscle, STIM1-R304Q was expressed in mouse primary skeletal myotubes, and the properties of the skeletal myotubes were examined using single-myotube Ca^2+^ imaging, transmission electron microscopy (TEM), and biochemical approaches. STIM1-R304Q did not interfere with the terminal differentiation of skeletal myoblasts to myotubes and retained the ability of STIM1 to attenuate dihydropyridine receptor (DHPR) activity. STIM1-R304Q induced hyper-SOCE (that exceeded the SOCE by wild-type STIM1) by affecting both the amplitude and the onset rate of SOCE. Unlike that by wild-type STIM1, hyper-SOCE by STIM1-R304Q contributed to a disturbance in Ca^2+^ distribution between the cytosol and the sarcoplasmic reticulum (SR) (high Ca^2+^ in the cytosol and low Ca^2+^ in the SR). Moreover, the hyper-SOCE and the high cytosolic Ca^2+^ level induced by STIM1-R304Q involve changes in mitochondrial shape. Therefore, a series of these cellular defects induced by STIM1-R304Q could induce deleterious skeletal muscle phenotypes in human patients carrying STIM1-R304Q.

## 1. Introduction

Initiation of skeletal muscle contraction is mediated by excitation–contraction (EC) coupling [1,2,3]. In short, t-tubule membrane depolarization serially activates (1) the dihydropyridine receptor (DHPR) on the t-tubule membrane, (2) the ryanodine receptor 1 (RyR1) on the sarcoplasmic reticulum (SR) membrane (by physical interactions between active DHPR and RyR1), and (3) Ca^2+^ release from the SR to the cytosol through active RyR1. Finally, Ca^2+^ in the cytosol turns on a series of contractile proteins by binding to troponin C. Therefore, the change in intracellular Ca^2+^ levels is a messenger event that connects membrane depolarization to muscle contraction during skeletal muscle contraction. In addition to the initiation of skeletal muscle contraction, the maintenance of high cytosolic Ca^2+^ levels during skeletal muscle contractions, such as long periods or repetitive contractions, is another important issue to understand skeletal muscle contraction.

Extracellular Ca^2+^ entry contributes to the maintenance of high cytosolic Ca^2+^ levels during skeletal muscle contractions, and store-operated Ca^2+^ entry (SOCE) is the main extracellular Ca^2+^ entry method in skeletal muscle [2,3]. Orai1 (a Ca^2+^ entry channel) on the t-tubule membrane and stromal interaction molecule 1 (STIM1, a Ca^2+^ sensor) on the SR membrane are the main SOCE-mediating proteins in skeletal muscle by the formation of puncta (i.e., oligomeric complexes of STIM1s and Orai1s). Various mutations in STIM1 (at H72, N80, G81, D84, S88, L92, L96, Y98, F108, H109, I115, E136X, P165, L251, R304, R426, R429, and I484) have been reported [4,5,6,7,8]. Patients with skeletal muscle diseases involving STIM1 mutations have also been reported [2,3,4,5,6,7,8]. For example, congenital myopathies have been associated with E136X; muscular hypotonia with R429C; tubular aggregate myopathy with N80T, G81D, L96V, F108I, H109R, I115F, or I484R; skeletal muscle atrophy and progressive muscle weaknesses with H72Q, D84G, H109N, H109R, or R304W.

STIM1 R304 is located in a coiled-coil domain of STIM1, and human patients with substitution of the arginine at residue 304 by tryptophan (STIM1-R304W, a constitutively active form of STIM1) show Stormorken syndrome, which is a multisystemic disease characterized by skeletal muscle phenotypes, including tubular aggregate myopathy, muscle spasms, muscle weakness, atrophy, lack of endurance, and hematological phenotypes [5,9,10,11]. Studies on STIM1-R304W with cells from patients with Stormorken syndrome or model mice carrying STIM1-R304W suggest that excessive SOCE is a cause of multisystemic defects caused by STIM1-R304W [4,5,12,13].

Interestingly, patients with substitution of the arginine at residue 304 by the less hydrophobic glutamine (STIM1-R304Q, another constitutively active form of STIM1) rather than tryptophan (STIM1-R304W) manifest a milder and lesser deleterious clinical phenotype of Stormorken syndrome, such as muscle weakness, myalgia, muscle stiffness, and contractures [4,9,10]. However, despite these clear symptoms in the skeletal muscle of patients with STIM1-R304Q [4,5,9,10], studies on pathological mechanisms of STIM1-R304Q have been conducted using only “nonmuscle cells”, such as fibroblasts or a heterologous expression system (HEK293 cells) [5,10]. Therefore, in this study, we aimed to reveal the pathological role(s) of STIM1-R304Q in skeletal muscle at the cellular level using mouse primary skeletal myotubes (instead of a heterologous expression system involving variations in the expression), single-myotube Ca^2+^ imaging experiments, and biochemical approaches.

## 2. Materials and Methods

### 2.1. Ethical Approval

The methods were carried out in accordance with the guidelines and regulations of the College of Medicine at the Catholic University of Korea. All surgical interventions, including pre- and postsurgical animal care and the site where the animal work was performed, were carried out in accordance with the Laboratory Animals Welfare Act, the Guide for Care and Use of Laboratory Animals, and the Guidelines and Policies for Rodent Survival Surgery approved by the Institutional Animal Care and Use Committee of the College of Medicine at the Catholic University of Korea. All experimental protocols were approved by the Committee of the College of Medicine at the Catholic University of Korea (2017-0117-01).

### 2.2. cDNA Construction, Cell Culture, and STIM1-R304Q Expression

The mutation of the R at residue 304 of STIM1 to Q (STIM1-R304Q) was carried out using human STIM1 cDNA as a template (GenBank accession number: NM_003156.3), a site-directed mutagenesis kit (Agilent Technologies, Santa Clara, CA, USA), and a pair of complementary synthetic oligonucleotide primers containing the desired mutation (forward primer, 5′-CGGCTGAAGGAGCTGCAGGAGGGTACTGAGAATG-3′; reverse primer, 5′-CATTCTCAGTACCCTCCTGCAGCTCCTTCAGCCG-3′) [7,8]. Mouse primary skeletal myoblasts were derived from mouse skeletal muscle using a single-cell cloning method, expanded, and differentiated into myotubes, as previously described [7,8,14,15]. After three days of culture under differentiation conditions, premature myotubes were transfected with an empty vector or cDNA encoding wtSTIM1 or STIM1-R304Q for 3 h. Mature myotubes were imaged, observed, or disrupted at 36 h posttransfection for further experiments. All reagents for the cell cultures were obtained from Invitrogen (Thermo Fisher Scientific, Waltham, MA, USA).

### 2.3. Immunocytochemistry and Immunoblot Assays

For immunocytochemistry assays, myotubes were fixed in cold methanol (−20 °C) for 30 min and permeabilized with 0.05% Tween 20 in PBS for 1 min, as previously described [7,8,16,17]. For immunoblot assays, solubilized lysate of myotubes (15 μg of total protein) was subjected to SDS–PAGE (8 or 10% gel), as previously described [7,8,16,17,18,19,20]. Anti-RyR1 and anti-SERCA1a antibodies were obtained from Affinity BioReagents. Anti-DHPR, anti-Orai1, anti-STIM1, and anti-α-actin antibodies were obtained from Abcam (Cambridge, MA, USA).

### 2.4. Single-Myotube Ca^2^^+^ Imaging

Single-myotube Ca^2^^+^ imaging was performed using an inverted-stage microscope (Nikon Eclipse TS100, Nikon Instruments, Inc., Melville, NY, USA) and a high-speed monochromator with a 75 W xenon lamp (FSM150Xe, Bentham Instruments, Reading, Berkshire, UK), as previously described [7,8,16,17,18,20]. The data were analyzed using image acquisition and analysis software (High-Speed InCyt Im1 and Im2, v5.29, Intracellular Imaging Inc., Cincinnati, OH, USA). Slopes at the rising phase of SOCE were examined by a linear equation, as previously described [7]. Reagents for single-myotube Ca^2^^+^ imaging were obtained from Sigma-Aldrich (St. Louis, MO, USA).

### 2.5. Transmission Electron Microscopy (TEM) Observation, Myotube Width Measurement, and Mitochondrial Length Measurement

Myotubes were fixed, embedded in epoxy resin (Epon 812), sectioned (70–80 nm) using an ultramicrotome (Ultracut UCT ultramicrotome, Leica, Buffalo Grove, IL, USA), and examined under TEM (JEM1010, JEOL Ltd., Peabody, MA, USA), as previously described [7,16]. Myotube width measurements or mitochondrial length measurements were performed using ImageJ software, as previously described [7,8,16,17,20].

### 2.6. Statistical Analysis

The results are presented as the mean ± SE for the number of myotubes shown in parentheses in the figure legends or tables. Group differences were analyzed using an unpaired *t*-test (GraphPad InStat, v2.04, GraphPad Software, San Diego, CA, USA). The differences were considered to be significant at *p* < 0.05. The graphs were prepared using Origin 2019b (OriginLab, Northampton, MA, USA).

## 3. Results and Discussion

### 3.1. By Affecting Both the Amplitude and the Onset Rate of SOCE, STIM1-R304Q Induces Hyper-SOCE in Skeletal Myotubes

**Figure 1:**
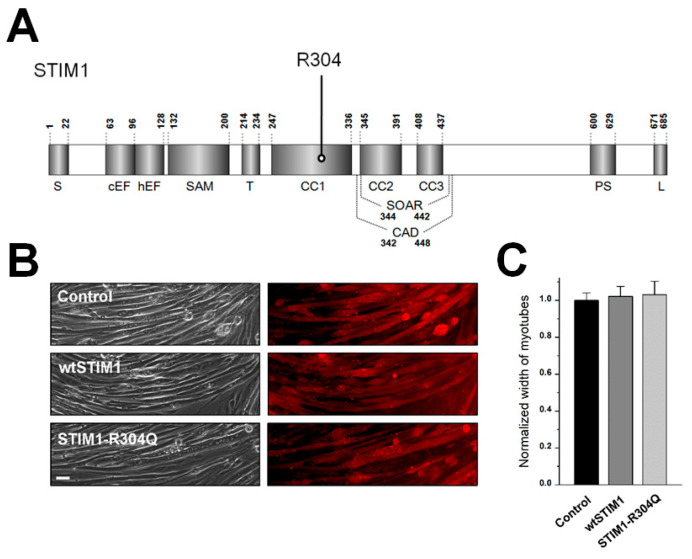
Schematic of the primary structure of STIM1, the expression of wtSTIM1 or STIM1-R304Q in mouse primary skeletal myotubes, and myotube width: (**A**) the location of R304 is indicated. Numbers indicate the amino acid sequence. Each domain of STIM1 is present [7]. S, signal peptide; cEF, canonical EF-hand; hEF, nonfunctional hidden EF-hand; SAM, sterile α-motif; T, transmembrane domain; CC, coiled-coil domain; CAD/SOAR, Ca^2^^+^ release-activated Ca^2^^+^-activating domain/STIM1-Orai1-activating region; PS, proline/serine-rich domain; and L, lysine-rich domain; (**B**) mouse primary skeletal myotubes that were transfected with cDNA of empty vector (control), wtSTIM1, or STIM1-R304Q were stained with anti-GFP (for detecting CFP or CFP-tagged proteins) and Cy3-conjugated secondary antibodies. The bar represents 100 μm; (**C**) myotube width was measured. The mean values of each normalized to the mean value of the control are summarized as histograms (Table 1).

To study the pathological mechanism of STIM1-R304Q in skeletal muscle (Figure 1A), wild-type STIM1 (wtSTIM1) or STIM1-R304Q was expressed in mouse primary skeletal myotubes (Figure 1B). Myotubes that were transfected with empty vectors were used as a control (also for subsequent experiments). Myotube width (one criterion that is used to evaluate the degree of terminal differentiation in skeletal muscle) was measured (Figure 1C). No significant difference was induced in myotube width by the expression of wtSTIM1 or STIM1-R304Q. This suggests that the expression of STIM1-R304Q does not significantly affect the terminal differentiation of skeletal muscle and again confirms that STIM1 is not a critical protein for the terminal differentiation of skeletal muscle [7,8,17]. Human patients carrying STIM1-R304W (another mutant at R304) show skeletal muscle atrophy [11]. Based on no significant change in myotube width by STIM1-R304Q, it seems that the atrophy induced by STIM1-R304W may not be due to the decrease in the width of each muscle fiber but to a reduction in the total number of muscle fibers. Indeed, model mice carrying STIM1-R304W show no significant change in the diameter of muscle fibers (a slight reduction or no change in male or female tibialis anterior muscle fibers, respectively) [21].

**Figure 2:**
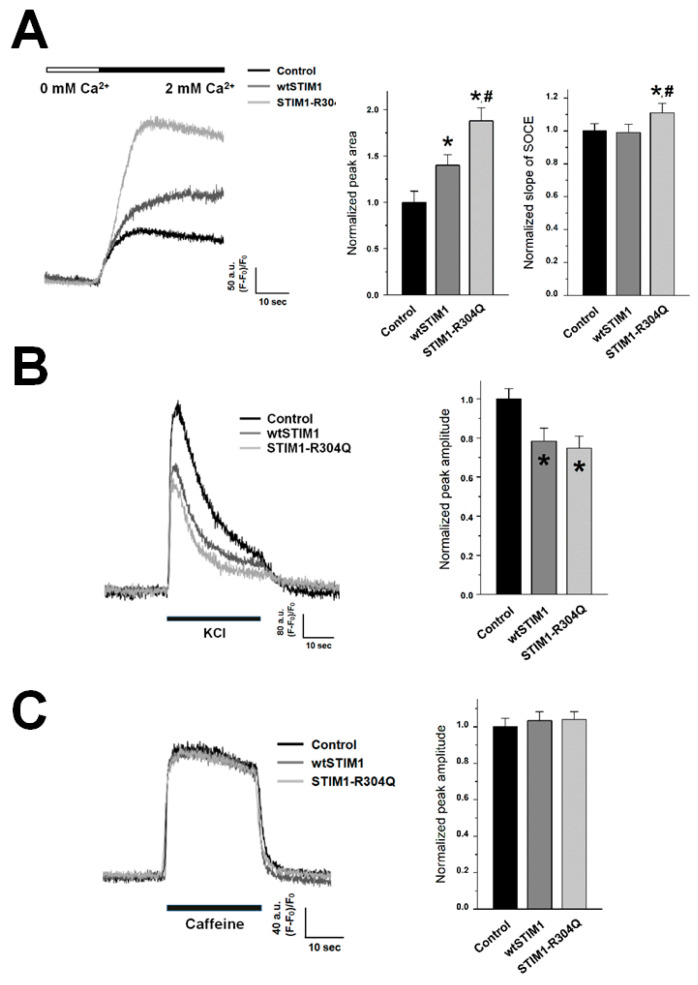
Ca^2+^ entry through the SOCE mechanism and intracellular Ca^2+^ release: (**A**) Ca^2+^ of the SR in wtSTIM1- or STIM1-R304Q-expressing myotubes was depleted by treatment with thapsigargin (2.5 μM) in the absence of extracellular Ca^2+^, and extracellular Ca^2+^ (2 mM) was then applied to the myotubes to induce SOCE. The experimental mean values were normalized to the mean values of the control (for the area under the peaks on the left-hand side and for the slope in the rising phase of SOCE on the right-hand side, Table 1). KCl (**B**) or caffeine (**C**) was applied to the myotubes, and intracellular Ca^2+^ release from the SR to the cytosol through RyR1 was measured. The experimental values were normalized to the mean values of the control (Table 1). A representative trace for each group is shown (**A**–**C**). * Significant difference compared with the control (*p* < 0.05). ^#^ Significant difference compared with wtSTIM1 (*p* < 0.05).

To examine the effect of STIM1-R304Q on SOCE in skeletal muscle, Ca^2^^+^ in the SR of STIM1-R304Q-expressing myotubes was depleted with thapsigargin in the absence of extracellular Ca^2^^+^ to prevent extracellular Ca^2^^+^ entry during depletion, and extracellular Ca^2^^+^ was then applied to the myotubes to measure SOCE (Figure 2A). As previously reported [7,8,17], wtSTIM1 significantly enhanced SOCE, compared with the control. STIM1-R304Q also significantly enhanced SOCE, compared with the control, which is in accordance with previous findings that were obtained from nonmuscle cells from patients with STIM1-R304Q (fibroblasts) or a heterologous expression system (HEK293 cells) [5,10]: the constitutively active property of STIM1-R304Q contributes to the enhancement of SOCE by STIM1-R304Q. The constitutively active property of STIM1-R304Q was confirmed in STIM1-R304Q-expressing myotubes (i.e., basal SOCE without the depletion of the SR by STIM1-R304Q, Supplementary Figure S1 and Table S1). Interestingly, compared with wtSTIM1, STIM1-R304Q also significantly enhanced SOCE (thus referred to as “hyper-SOCE” in the present study). Considering that homo-hexamerization of STIM1 is required to evoke SOCE [2,22], it is very likely that STIM1-R304Q could form hetero-hexamers with endogenous STIM1s. Therefore, the ability of STIM1-R304Q to induce hyper-SOCE could be underestimated in this experimental condition, and SOCE in the skeletal muscle fibers from the human patients carrying STIM1-R304Q could be much more severely intensified than the hyper-SOCE in the present study.

In addition, STIM1-R304Q-expressing myotubes showed another property: the slope at the rising phase of SOCE was significantly steeper (i.e., faster onset of SOCE) than that in control- or wtSTIM1-expressing myotubes (Figure 2A, right histograms). These results suggest that STIM1-R304Q induces hyper-SOCE by affecting both the amplitude and the onset rate of SOCE. Therefore, the working mechanisms of wtSTIM1 (via the formation of puncta with Orai1, as mentioned in the Introduction section), and STIM1-R304Q in enhancing SOCE could be different.

It has been reported that the mutation of the R at residue 304 to W could destabilize the conformation of STIM1-R304W and promote the exposure of its Orai1-binding site (known as the CAD/SOAR domain) to form permanent puncta with Orai1s and subsequently excessive SOCE [5]. Considering that the substitution mutations, STIM1-R304W and STIM1-R304Q, show the same tendency (i.e., a substitution of a positively charged amino acid (R) to an amino acid with an uncharged side chain (W or Q)), the way that STIM1-R304W induces excess SOCE could also be possible for STIM1-R304Q. In addition, it has been reported that STIM1-R304W could also enhance SOCE via the suppression of Orai1 inactivation [23].

### 3.2. STIM1-R304Q Retains the Ability of STIM1 to Attenuate DHPR Activity in Skeletal Myotubes

Based on reports that STIM1 attenuates DHPR (Ca_V_1.1) activity during EC coupling in skeletal muscle [7,8] and that STIM1 suppresses Ca_V_1.2 activity in T lymphocytes [24], a membrane depolarizer (KCl that induces intracellular Ca^2^^+^ release by inducing membrane depolarization and activating DHPR, such as during EC coupling [7,8,17]) was applied to STIM1-R304Q-expressing myotubes, and intracellular Ca^2^^+^ release from the SR to the cytosol through RyR1 was measured using single-myotube Ca^2^^+^ imaging (Figure 2B). As shown in our previous findings [7,8,17], wtSTIM1 significantly decreased intracellular Ca^2^^+^ release in response to membrane depolarization, compared with the control, and STIM1-R304Q showed the same phenomenon. These results suggest that the ability of STIM1-R304Q to attenuate DHPR activity is indistinguishable from that of wtSTIM1.

To rule out the possibility that the decrease in intracellular Ca^2^^+^ release in response to membrane depolarization in STIM1-R304Q-expressing myotubes in Figure 2B was simply due to a decrease in RyR1 activity rather than the attenuation of DHPR activity by STIM1-R304Q, RyR1 activity was assessed by applying caffeine (a direct agonist of RyR1 [25]) to STIM1-R304Q-expressing myotubes (Figure 2C). There was no significant change in intracellular Ca^2^^+^ release in response to caffeine by either wtSTIM1 or STIM1-R304Q compared with the control, suggesting that the overall activity of RyR1 was not changed by either one.

### 3.3. STIM1-R304Q Disturbs the Intracellular Ca^2+^ Distribution between the Cytosol and the SR in Skeletal Myotubes

**Figure 3:**
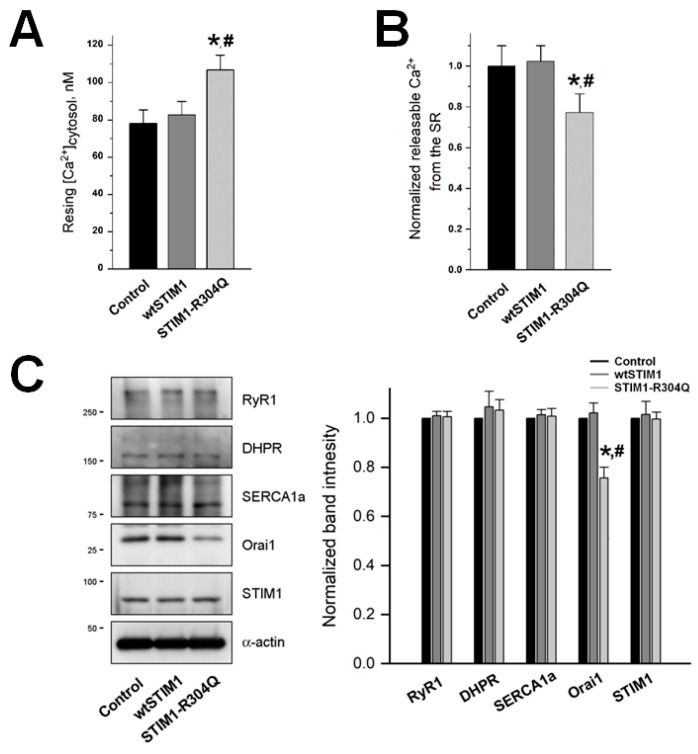
Cytosolic Ca^2+^ levels, the amount of Ca^2+^ releasable from the SR, and expression levels of Ca^2+^ movement-mediating proteins: (**A**) cytosolic Ca^2+^ levels at rest were measured in wtSTIM1- or STIM1-R304Q-expressing myotubes, and the mean values are summarized as histograms (Table 1); (**B**) amount of Ca^2+^ releasable from the SR to the cytosol was measured in the myotubes by treatment with thapsigargin (2.5 μM) in the absence of extracellular Ca^2+^. The mean values of each normalized to the mean value of the control are summarized as histograms (Table 1); (**C**) the lysate of the myotubes was subjected to immunoblot assays with antibodies against five proteins. *α*-actin was used as a loading control. The expression level of each protein normalized to the value of each control is presented as histograms (Supplementary Table S2). * Significant difference compared with control (*p* < 0.05). ^#^ Significant difference compared with wtSTIM1 (*p* < 0.05).

To examine other effects of STIM1-R304Q in skeletal muscle, first, cytosolic Ca^2^^+^ levels at rest were measured in STIM1-R304Q-expressing myotubes (Figure 3A). The cytosolic Ca^2^^+^ level was not significantly changed by wtSTIM1, compared with the control, as was previously found [7,8,17]. However, STIM1-R304Q significantly increased cytosolic Ca^2^^+^ levels. Second, the amount of Ca^2^^+^ releasable from the SR (which allows an estimation of Ca^2^^+^ level in the SR) was measured by depleting the SR with thapsigargin in the absence of extracellular Ca^2^^+^ to avoid extracellular Ca^2^^+^ entry during SR depletion (Figure 3B). Interestingly, unlike wtSTIM1 with no change, STIM1-R304Q significantly decreased Ca^2^^+^ levels in the SR. It is likely that in STIM1-R304Q-expressing myotubes, the low Ca^2^^+^ level in the SR could contribute to the high Ca^2^^+^ level in the cytosol (i.e., a disturbance in intracellular Ca^2^^+^ distribution between the cytosol and the SR). Considering that STIM1-R304Q induced hyper-SOCE, it is certain that hyper-SOCE induced by STIM1-R304Q also contributes to the high cytosolic Ca^2^^+^ level in STIM1-R304Q-expressing myotubes. On the other hand, it is possible that the decrease in intracellular Ca^2^^+^ release in response to membrane depolarization in STIM1-R304Q-expressing myotubes in Figure 2B could be used as a compensatory mechanism for the further Ca^2^^+^ decrease in the SR (i.e., to not lose any more Ca^2^^+^ from the SR) and the further Ca^2^^+^ increase in the cytosol (i.e., to not release any more Ca^2^^+^ to the cytosol). Otherwise, the disturbance in Ca^2^^+^ distribution between the cytosol and the SR by STIM1-R304Q could be much intensified.

To assess the expression levels of the main proteins that mediate Ca^2^^+^ movements during EC coupling or SOCE in skeletal muscle, immunoblot assays were conducted with lysates of STIM1-R304Q-expressing myotubes (Figure 3C). There was no significant change in the expression levels of RyR1, DHPR, SERCA1a, or endogenous STIM1. The expression level of Orai1 was even significantly decreased (possibly to compensate for the hyper-SOCE by STIM1-R304Q). Junctophilins (JP1 and JP2) are important contributors to the formation and maintenance of junctional membrane complex in skeletal muscle [2,3]. The expression levels of JP1 and JP2 in STIM1-R304Q-expressing myotubes were also assessed. There was no significant change in their expression levels (Supplementary Figure S2; Table S3). These results suggest that changes in SOCE, intracellular Ca^2^^+^ release during EC coupling, and intracellular Ca^2^^+^ distribution in STIM1-R304Q-expressing myotubes in Figure 2 and Figure 3 were not caused by a change in the expression level of the proteins but were instead due to the mutation at R304.

On the other hand, transient receptor potential canonical proteins (TRPCs) are also known as SOCE channels in skeletal muscle [26]. Among four types of TRPCs that are expressed in skeletal muscle, expression levels of TRPC1 and TRPC3 (that are dominantly expressed types) were examined by immunoblot assays (Supplementary Figure S2; Table S3). Unlike TRPC3, the expression level of TRPC1 was significantly increased in STIM1-R304Q-expressing myotubes, suggesting a possible clue that, in addition to Orai1, TRPC1 could contribute to the hyper-SOCE by STIM1-R304Q.

### 3.4. STIM1-R304Q Changes the Shape of Mitochondria in Skeletal Myotubes

**Figure 4:**
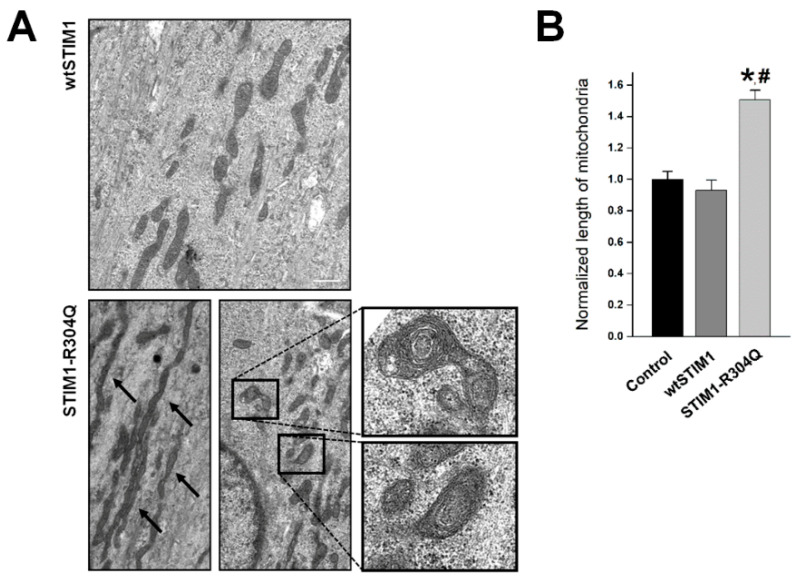
Shapes of mitochondria: (**A**) mitochondria of wtSTIM1- or STIM1-R304Q-expressing myotubes were observed using TEM. Mitochondria with concentrically laminated bodies (enlarged images) or long mitochondria (indicated by arrows) were found in STIM1-R304Q-expressing myotubes. The bar represents 2 μm; (**B**) mitochondrial length was measured. The mean values of each normalized to the mean value of the control are summarized as histograms (Table 1). * Significant difference compared with the control (*p* < 0.05). ^#^ Significant difference compared with wtSTIM1 (*p* < 0.05).

To assess the aftereffects of hyper-SOCE and the disturbance in intracellular Ca^2^^+^ distribution by STIM1-R304Q, STIM1-R304Q-expressing myotubes were observed by TEM (Figure 4A). First, mitochondria with concentrically laminated bodies were found in STIM1-R304Q-expressing myotubes (approximately 15% of the total mitochondria, enlarged images in Figure 4A). Mitochondria with concentrically laminated bodies have been reported in the skeletal and cardiac muscle of human patients with cardiomyopathy and chronic congestive heart failure [27]. Cytosolic Ca^2^^+^ is one of the master regulators of mitochondrial biogenesis [28]. Therefore, it is possible that the high cytosolic Ca^2^^+^ level induced by STIM1-R304Q in Figure 3A could disrupt the signaling for mitochondrial biogenesis and induce the change in mitochondrial shape. Similarly, model mice with STIM1-R304W show swollen mitochondria along with excessive SOCE [11]; however, the link between swollen mitochondria and excessive SOCE has not been addressed. The present study suggests a possible link that a high intracellular Ca^2^^+^ level due to excess or hyper-SOCE and less Ca^2^^+^ in the SR could induce abnormal shapes in mitochondria, such as concentric laminated bodies or swelling.

Second, mitochondrial length was significantly increased in STIM1-R304Q-expressing myotubes (Figure 4B). The exercise state is more favorable to form long mitochondria, and fragmented mitochondria are usually more dominant in aging, atrophy, disuse, or disease states [28]. Therefore, it is possible that to compensate for the possibly malfunctioning mitochondria with concentrically laminated bodies, long mitochondria could exist by playing a protective role in STIM1-R304Q-expressing myotubes.

This study was focused on the pathological mechanism of STIM1-R304Q (which is a gain-of-function mutant of STIM1 (i.e., hyper-SOCE) that causes muscle weakness, myalgia, muscle stiffness, and contractures). In our previous study, the pathological property of R429C (which is a loss-of-function mutant of STIM1 (i.e., null-SOCE) and causes human muscular hypotonia) was examined [7]. Surprisingly, these two opposite mutants, in terms of mediating SOCE, have similar characteristics in their tendency in intracellular Ca^2^^+^ release during skeletal muscle contraction, cytosolic Ca^2^^+^ levels, the amount of Ca^2^^+^ releasable from the SR, myotube width, and abnormal mitochondria, with the notable exception of SOCE. These two independent studies on STIM1 strongly suggest that if abnormal SOCE occurs as a result of STIM1 mutants in skeletal muscle (either way of abnormal extracellular Ca^2^^+^ entry from null to hyper-SOCE), similar adaptive mechanisms to alleviate the effects of the abnormal SOCE could occur to prevent further functional defects in skeletal muscle and subsequent systemic defects.

## Figures and Tables

**Table 1:** Properties of wtSTIM1 or R340Q-expressing mouse primary skeletal myotubes. The values, except for those of the cytosolic Ca^2+^ levels at rest, were normalized to the mean value of those from the control. The values are presented as the mean ± SE for the number of myotubes shown in parentheses. * Significant difference compared with control (*p* < 0.05). ^#^ Significant difference compared with wtSTIM1 (*p* < 0.05).

		Control	wtSTIM1	STIM1-R304Q
Width of myotubes		1.00 ± 0.04 (50)	1.02 ± 0.05 (50)	1.03 ± 0.07 (50)
SOCE	Peak area	1.00 ± 0.12 (40)	1.40 ± 0.11 * (40)	1.88 ± 0.14 *, ^#^ (40)
	Slope	1.00 ± 0.05 (30)	0.99 ± 0.05 (30)	1.11 ± 0.06 *^, #^ (30)
KCl response		1.00 ± 0.05 (70)	0.78 ± 0.07 * (70)	0.75 ± 0.06 * (70)
Caffeine response		1.00 ± 0.05 (70)	1.03 ± 0.05 (70)	1.04 ± 0.04 (70)
Resting [Ca^2+^]cytosol, nM		78.16 ± 7.07 (50)	82.73 ± 7.14 (50)	106.76 ± 7.94 *^, #^ (50)
Amount of Ca^2+^ releasable from the SR		1.00 ± 0.11 (40)	1.02 ± 0.08 (40)	0.77 ± 0.09 *^, #^ (40)
Length of mitochondria		1.00 ± 0.05 (62)	0.93 ± 0.07 (61)	1.51 ± 0.06 *^, #^ (69)

## Supplementary Materials

The following are available online at https://www.mdpi.com/article/10.3390/biom11081064/s1, Table S1: Expression levels of proteins that mediate Ca^2+^ movement in wtSTIM1 or STIM1-R340Q-expressing myotubes. Table S2: Expression levels of proteins that mediate Ca^2+^ movement in wtSTIM1 or STIM1-R340Q-expressing myotubes. Table S3: Expression levels of JP1, JP2, TRPC1, or TRPC3 in wtSTIM1 or STIM1-R340Q-expressing myotubes. Figure S1: Extracellular Ca^2+^ entry without the depletion of the SR in wtSTIM1 or STIM1-R340Q-expressing myotubes. Figure S2: Expression levels of JP1, JP2, TRPC1, or TRPC3 in wtSTIM1 or STIM1-R340Q-expressing myotubes.

## Abbreviations

STIM1	Stromal interaction molecule1
SOCE	Store-operated Ca^2+^ entry
EC	Excitation–contraction
DHPR	Dihydropyridine receptor
RyR1	Ryanodine receptor 1
SERCA1a	Sarcoplasmic/endoplasmic reticulum Ca^2+^-ATPase 1a
