# Supplementary material for: Pathological Mechanism of a Constitutively Active Form of Stromal Interaction Molecule 1 in Skeletal Muscle
Source: Biomolecules. 2021 Jul 21;11(8):1064. doi: 10.3390/biom11081064 (PMC8394508; doi:10.3390/biom11081064)
Supplement: Supplementary file 1 [file biomolecules-11-01064-s001.zip › biomolecules-1276659-supplementary.pdf]

Pathological Mechanism of a Constitutively Active Form of Stromal Interaction Molecule 1 in Skeletal Muscle

List of Supplementary Materials

Supplementary Figure S1. Extracellular Ca^{2+} entry without the depletion of the SR in wtSTIM1 or STIM1-R340Q-expressing myotubes

Supplementary Figure S2. Expression levels of JP1, JP2, TRPC1, or TRPC3 in wtSTIM1 or STIM1-R340Q-expressing myotubes

Supplementary Table S1. Extracellular Ca^{2+} entry without the depletion of the SR in wtSTIM1 or STIM1-R340Q-expressing myotubes

Supplementary Table S2. Expression levels of proteins that mediate Ca^{2+} movement in wtSTIM1 or STIM1-R340Q-expressing myotubes

Supplementary Table S3. Expression levels of JP1, JP2, TRPC1, or TRPC3 in wtSTIM1 or STIM1-R340Q-expressing myotubes

Supplementary Figure S1

Extracellular Ca^{2+} entry without the depletion of the SR in wtSTIM1 or STIM1-R340Q-expressing myotubes. (A) Extracellular Ca^{2+} entry without the depletion of the SR (i.e., basal SOCE) in wtSTIM1- or STIM1-R304Q-expressing myotubes was measured. The myotubes were incubated with the imaging solution without Ca^{2+} (0 mM) for 15 min, and extracellular Ca^{2+} (2 mM) was then applied to the myotubes to induce basal SOCE. A representative trace for each group is shown. **(B)** The experimental mean values were normalized to the mean values of the control (for the area under the peaks of basal SOCE) and the values are presented as the mean ± SE for the number of myotubes shown in parentheses of Supplementary Table S1. *Significant difference compared with control ($p < 0.05$). #Significant difference compared with wtSTIM1 ($p < 0.05$).

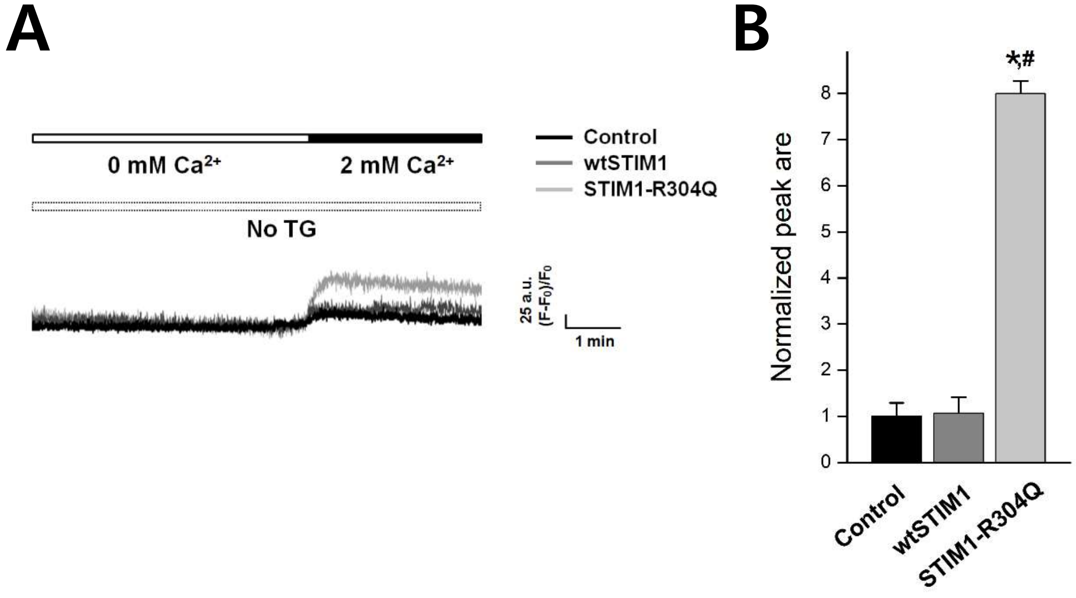

Supplementary Figure S2

Expression levels of JP1, JP2, TRPC1, or TRPC3 in wtSTIM1 or STIM1-R340Q-expressing myotubes. (A) Lysate of wtSTIM1- or STIM1-R304Q-expressing myotubes was subjected to immunoblot assays with one of the antibodies against JP2, JP2, TRPC1, or TRPC3. α-actin was used as a loading control. **(B)** The expression level of each protein normalized to the value of each control is presented as histograms (Supplementary Table S3). Three independent experiments were conducted per each protein. *Significant difference compared with control (p < 0.05). #Significant difference compared with wtSTIM1 (p < 0.05).

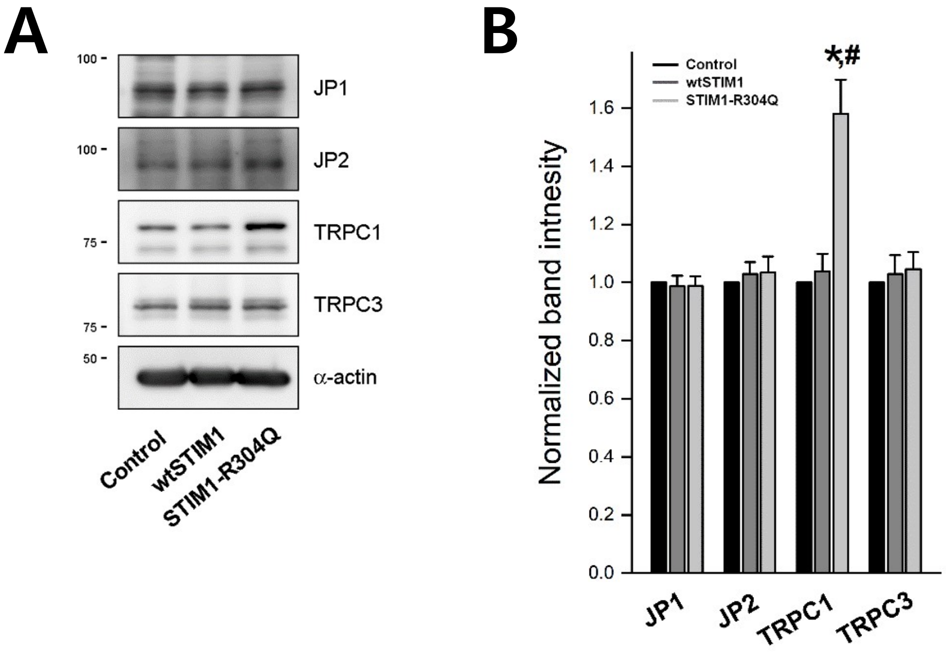

Supplementary Table S1

Extracellular Ca^{2+} entry without the depletion of the SR in wtSTIM1 or STIM1-R340Q-expressing myotubes. Extracellular Ca^{2+} entry without the depletion of the SR (i.e., basal SOCE) in wtSTIM1- or STIM1-R304Q-expressing myotubes was measured. The myotubes were incubated with the imaging solution without Ca^{2+} (0 mM) for 15 min, and extracellular Ca^{2+} (2 mM) was then applied to the myotubes to induce basal SOCE. The experimental mean values were normalized to the mean values of the control (for the area under the peaks of basal SOCE) and the values are presented as the mean ± SE for the number of myotubes shown in parentheses. *Significant difference compared with control ($p < 0.05$). #Significant difference compared with wtSTIM1 ($p < 0.05$).

	**Control**	**wtSTIM1**	**STIM1-R304Q**
**Basal SOCE**	1.00 ± 0.29<br>(47)	1.07 ± 0.35<br>(51)	8.00 ± 0.27 *, #<br>(50)

Supplementary Table S2

Expression levels of proteins that mediate Ca^{2+} movements in wtSTIM1 or STIM1-R340Q-expressing myotubes. The lysate of wtSTIM1- or STIM1-R304Q-expressing myotubes was subjected to immunoblot assays with one of the antibodies against five proteins that mediate intracellular Ca^{2+}-release or SOCE in skeletal muscle. α-actin was used as a loading control. Three independent experiments were conducted per each protein. *Significant difference compared with the control ($p < 0.05$). #Significant difference compared with wtSTIM1 ($p < 0.05$).

	**Control**	**wtSTIM1**	**STIM1-R304Q**
**RyR1**	1.00 ± 0.00	1.01 ± 0.02	1.01 ± 0.02
**DHPR**	1.00 ± 0.00	1.05 ± 0.06	1.03 ± 0.04
**SERCA1a**	1.00 ± 0.00	1.01 ± 0.02	1.01 ± 0.03
**Orai1**	1.00 ± 0.00	1.02 ± 0.04	0.76 ± 0.04 *, #
**STIM1**	1.00 ± 0.00	1.01 ± 0.05	1.00 ± 0.03

Supplementary Table S3

Expression levels of JP1, JP2, TRPC1, or TRPC3 in wtSTIM1 or STIM1-R340Q-expressing myotubes. The lysate of wtSTIM1- or STIM1-R304Q-expressing myotubes was subjected to immunoblot assays with one of the antibodies against JP1, JP2, TRPC1, or TRPC3. *α*-actin was used as a loading control. Three independent experiments were conducted per each protein. *Significant difference compared with the control ($p < 0.05$). #Significant difference compared with wtSTIM1 ($p < 0.05$).

	**Control**	**wtSTIM1**	**STIM1-R304Q**
**JP1**	1.00 ± 0.00	0.99 ± 0.04	1.03 ± 0.04
**JP2**	1.00 ± 0.00	0.99 ± 0.03	1.03 ± 0.05
**TRPC1**	1.00 ± 0.00	1.04 ± 0.06	1.58 ± 0.12 *, #
**TRPC3**	1.00 ± 0.00	1.03 ± 0.06	1.05 ± 0.06
